# Herpes zoster-associated mortality in Europe: a systematic review

**DOI:** 10.1186/s12889-015-1753-y

**Published:** 2015-05-05

**Authors:** Hélène Bricout, Margaret Haugh, Olugbenga Olatunde, Ruth Gil Prieto

**Affiliations:** Epidemiology Department, Sanofi Pasteur MSD, 162 avenue Jean Jaurès, CS 50712, 69367 Lyon cedex 07, France; MediCom Consult, 39 rue Clement Michut, 69100 Villeurbanne, France; Life Events and Population Sources, Office for National Statistics, Government Buildings, Newport, NP10 8XG UK; Department of Preventive Medicine and Public Health, Rey Juan Carlos University, Avda. Atenas s/n, CP: 28922, Alcorcón, Madrid Spain

**Keywords:** Herpes zoster, Varicella-zoster virus, Mortality, Europe

## Abstract

**Background:**

Reactivation of latent varicella zoster virus, partly due to age-related immunosenescence and immunosuppressive conditions, results in herpes zoster (HZ) and its associated complications. The management of the most important complication, post-herpetic neuralgia (PHN), is challenging, particularly in the elderly, and is generally unsatisfactory. No previous reviews have reported the incidence of HZ-associated mortality.

**Methods:**

We carried out a systematic literature review to identify studies and databases providing data for HZ-associated mortality in adults aged ≥50 years in Europe.

**Results:**

We identified 12 studies: Belgium (1); France (1); Germany (1); the Netherlands (2); Portugal (1); Spain (4) and England/Wales (2) and 4 databases from Europe: France; Germany and England/Wales. The incidence was available from eight studies; it was highest in those aged ≥95 in France (19.48/100,000). In the European (WHO) database, the overall mortality ranged from 0 to >0.07/100,000. The age- and gender-specific HZ mortality rates from the other databases showed that while in younger age groups the HZ mortality rate was higher in males, in older patients the rate was much higher in women. The case fatality rate was 2 and 61/100 000 in those 45–65 and ≥65 years, respectively. A similar increase with age was seen for the hospital fatality rate; 0.6% in those 45–65 years in the UK and 7.1% in those ≥80 in Spain.

**Conclusions:**

Although the data were sparse and heterogeneous, HZ-associated mortality clearly increases with age. In addition, the elderly who develop HZ often have underlying diseases and are at increased risk of functional decline and loss of independence. Mortality should be taken into account in health-economics models.

## Background

Varicella-zoster virus (VZV) is a herpes virus that infects nearly all humans and causes two distinct diseases: varicella, the primary infection which usually occurs in childhood, and herpes zoster (HZ) which is the result of the reactivation of VZV which remains latent in the sensory ganglia following primary infection. This reactivation occurs when VZV-specific cellular-mediated immunity decreases, mainly due to age-related immunosenescence and immunosuppressive conditions.

HZ is characterized by a vesicular skin rash localized in the sensory region of the affected ganglia, and is often preceded, or accompanied by acute pain or itching. The individual lifetime risk of developing HZ is between 24% and 30%, or approximately 1 in 4 people [1-5]. However, for individuals aged 85 and over, this risk increases to 1 in 2 [6]. HZ incidence increases markedly after 50 years of age, with two-thirds of HZ cases occurring in individuals aged 50 years and over [7]. Anyone who has had varicella is at risk of HZ; in Europe varicella affects over 90% of children before the age of 15 [8].

HZ is painful during the acute phase, but pain may persist for months or even years. Post-herpetic neuralgia (PHN), defined as chronic pain persisting after rash onset, occurs in 20% to 50% of patients, and can lead to several months of treatment and loss of quality of life [9,10]. After 1 year, almost 10% of patients, mainly older people, still have persistent pain [11,12].

A recent literature review showed that the annual HZ incidence is similar throughout Europe, varying from 2.0 to 4.6/1 000 person-years, with no clear geographic trend [13]. Age-specific HZ incidence rates are around 1/1 000 children <10 years, around 2/1 000 adults aged <40 years, and around 1–4/1 000 adults aged 40–50 years, increasing to around 7–8/1 000 after age 50 years, up to 10/1 000 after 80 years of age. This review confirmed that, in Europe, HZ incidence increases with age, particularly after 50 years of age [13]. Similarly, data on the percentage of HZ cases who develop PHN are available across EU [14]. PHN, defined as HZ-associated pain lasting for at least three months, has been reported to occur in 10% to 20% of patients. However, the prevalence and severity of PHN increases with age, and has been reported to be as high as 60% to 70% of patients aged ≥60 [14]. In people aged over 50 years, there is a higher rate of HZ-associated hospitalization and HZ and PHN have a greater impact on the quality of life of patients and their relatives.

To date, a review of the data for HZ-associated mortality has not been published. To fill this gap and to have a complete picture of the burden of HZ and PHN, we performed a systematic literature review of data on HZ-associated mortality in people aged ≥50 years in Europe.

## Methods

### Objectives

The objective of this systematic literature review was to summarize the available data on the HZ-associated mortality in adults aged ≥50 years in Europe.

### Search strategy

We searched PubMed from 1st January 1990 to 31 October 2013 using a combination of the following search terms as free text and medical subheading (MeSH) terms: mortality; death; case fatality rate; hospital fatality rate; Herpes Zoster [MeSh]; Herpes Zoster Ophthalmicus [MeSh]; Herpes Zoster Oticus [MeSh]; Encephalitis, Varicella Zoster [MeSh]; shingles, herpes zoster. We also scanned reference lists in selected publications to identify additional references, including publications known to the authors, not indexed on PubMed. No language restrictions were used.

In addition, European surveillance data, such as the European Detailed Mortality Database (DMDB) [15], national surveillance data, such as the CépiDc database from INSERM [16], the Office for National Statistics for England and Wales [17] and the German Federal Health Monitoring System [18] and the websites of the national health institutes and sentinel networks were searched.

The literature search was performed by one author (MH). The search results were downloaded into EndNote® for screening.

### Inclusion and exclusion criteria

We included publications or websites/databases that provided HZ-associated mortality data for adults aged ≥50 years, irrespective of their health status, in any of the following European Union (EU) countries: Austria; Belgium; Bulgaria; Croatia; Cyprus; Czech Republic; Denmark; Estonia; Finland; France; Germany; Greece; Hungary; Ireland; Italy; Latvia; Lithuania; Luxembourg; Malta; the Netherlands; Norway; Poland; Portugal; Romania; Slovakia; Slovenia; Spain; Sweden; Switzerland; and United Kingdom. Case reports were excluded.

The HZ-associated mortality data could include: total number of HZ deaths; HZ-mortality incidence; HZ case fatality rate (CFR) or hospital fatality rate (HFR). The CFR was defined as the percentage of those who died among community-based cases and the HFR as the percentage of those who died among hospitalized cases.

### Study selection process

**Figure 1:**
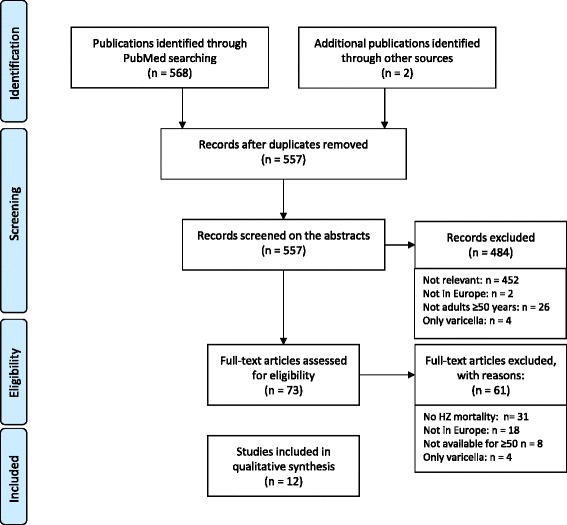
PRISMA diagram - summary of search results.

The first round of screening was performed on the titles and abstracts by one author (MH) and another author (HB) verified the included and excluded publications. Full papers were obtained for the second round of screening that was performed by one author (MH) and verified by another (HB). The reasons for exclusion from this screening round were recorded (using broad categories, e.g. data not available for adults >50; population outside the defined EU countries). The search results were summarised using the PRISMA flow diagram (Figure 1) [19]. Papers reporting HZ-associated mortality data for countries outside Europe were kept for comparisons and discussion*.*

### Quality assessment of included studies

As it was expected that there would not be many studies reporting HZ mortality rates, we decided to include all available data, without restrictions based on quality and to describe the quality of the data.

### Data analyses

The following data were extracted from the included studies by one author (MH) and verified by another author (HB): country, date, data source, HZ diagnosis, mean age at HZ related death, presence of comorbidities and HZ mortality related data. In some studies where the available data allowed for estimation, the CFR or HFR were calculated by dividing the number of deaths by the number of HZ cases in the community or by the number of patients hospitalized with HZ, respectively. In addition, in studies where only the annual number of death at the national level was available, the age-specific mortality rate was estimated by obtaining the country age-specific population from the EuroStats database [20].

## Results

### Data sources identified

A total of 568 references were identified on PubMed and 2 were identified by the authors (Figure 1). Following removal of duplicate publications, 557 abstracts were first screened on titles and abstracts and 484 references were excluded. Full papers were obtained for the remaining 73 references; 12 of these satisfied the inclusion criteria for this review. The reasons for non-inclusion were: no HZ-associated mortality data available (31); not European data (18); not possible to have data for adults aged ≥50 years (8); only varicella (4).

The 12 studies included were from 7 countries: Belgium (1); France (1); Germany (1); the Netherlands (2); Portugal (1); Spain (4) and England and Wales (2) (Table 1) [2,21-31]. In addition, we identified four databases that provided HZ-associated mortality data from Europe (1); France (1); Germany (1) and England and Wales (1) (Table 2) [15-18]. A number of other mortality databases were identified but it was not possible to extract HZ-specific mortality [32].

**Table 1:** **Characteristics of included studies**

**Study ID [ref]**	**Country/Dates**	**Data source**	**HZ diagnosis**	**Age (years)**	**Comorbidity**	HZ-associated mortality data available or calculated*
**Bilcke 2012** [21]	Belgium/1990-2007	National death certificates	Code for HZ as possible cause of death (no indication of primary or associated) and independent evaluation by 5 experts	≥60	None	Overall incidence
**Gonzalez Chiappe 2010** [27]	France/2000-2007	National death certificate database (INSERM CepiDC: http://www.cepidc.inserm.fr/)	ICD-10 codes B020.0-B020.9 as primary or associated cause of death	96.8% of deaths occurred in ≥65	21.6% had ≥1 underlying condition (59.6% malignancies, 12.5% diabetes, 10.9% blood dyscrasias and some immune deficiencies)	Age-specific incidence; disease-specific number of HZ deaths and hospital fatality rate*
**Ultsch 2011** [30]	Germany/2007-2008	Federal Health Monitoring database	ICD-10 codes of primary disease	≥50	None	Overall, and age- and gender-specific incidences and hospital fatality rate*
**van Lier 2010** [31]	Netherlands/2000-2007	National Statistics (CBS) database	ICD-10 codes B02 and G530 as primary cause of death	92% of deaths in ≥75	None	Number of HZ deaths; incidence of HZ mortality*
**de Melker 2006** [23]	Netherlands/1996-2002	National Statistics (CBS) database	ICD-10 code B02 as primary cause of death	97% in ≥60	None	Number of VZV deaths, incidence of HZ mortality*
**Mesquita 2013** [28]	Portugal/2000-2010	National Health Hospital Ministry Database	IDC-9 codes 053.10-13, 053.19-22, 053. 29, 053.71, 053.79, 053.8, 053.9 as primary diagnosis at hospital admission	All ages, with 10-year groups from 50 to 99	None	In-hospital fatality rate (not available by gender)
**Perez-Sola 2011** [29]	Spain/2000-2006	Registry for drug safety in rheumatic patients treated with anti-TNF (100 Spanish health centres)	Microbiological diagnosis	50 (mean; SD = 14)	Rheumatic diseases treated with TNF antagonists	Number of HZ deaths
**Gil 2009** [24]	Spain/1998-2004	National hospital discharge database (CMBD)	ICD-9 code for primary or secondary diagnosis: 0.53, 053.0-053.9	68.3 (SD = 15.5) for hospitalisations	None	Number of HZ deaths; age-specific hospital-mortality incidence; age-specific hospital fatality rate
**Gil-Prieto 2011** [26]	Spain/1998-2004	National hospital discharge database (CMBD)	ICD-9 code for primary or secondary diagnosis (053; 053.0-053.9)	>50	60.8% with diabetes, COPD and/or cardiovascular diseases, Exclusion of immunocompromised	Overall, age-specific and disease-specific hospital fatality rate
**Gil-Prieto 2012** [25]	Spain/1997-2008	National hospital discharge database (CMBD)	ICD-9-CM code for primary or secondary diagnosis for HZ ophthalmicus HZO 053.2, 053.20-22, 053.29	>50; 50–59; 60–69; >70	25% immunocompromised (all ages)	Number of HZO deaths; HZO overall hospital-fatality rate; HZO age specific hospital-fatality rate
					Comorbidities (cardiac chronic diseases, diabetes, COPD, asthma, chronic renal diseases)	
**Brisson 2003** [22]	England and Wales/1993-2000	Hospitalisation Episode Statistics database (national) & Office for National Statistics (death certificates)	ICD-10 code B02 as primary diagnosis and ICD Code for HZ as cause of death	45-64; 65+	8% with underlying conditions	Number of HZ deaths; Age-specific incidence; hospital-fatality rate
**Edmunds 2001** [2]	England and Wales/1998 (1985–94; 1995–1999)	Hospitalisation Episode Statistics database (national) & Office for National Statistics (death certificates)	ICD-10 code B02 in any of the 7 diagnosis fields/ICD-9 code 053 death (no details for primary or associated)	45+; and 5-year sub-groups	None	Age-specific mortality and case fatality rate

*to indicate that these have been calculated.

**Table 2:** **Characteristics of included surveillance systems/databases**

**Database name [Ref]**	**Region/dates covered**	**HZ diagnosis**	**HZ-associated mortality data available**
**WHO-DMDB** [15]	Europe/2010, except Belgium and France: 2009	ICD-10 code: B02 for primary cause of death	Age-specific and overall incidence by country
**CépiDc** [16]	France/2010	ICD-10 code: B02 for primary cause of death	Age-specific incidence
**Federal Health Monitoring System** [18]	Germany/2011 and previous years	ICD-10 code: B02 and sub-codes	Number and incidence for overall and sex by year
**Office for National Statistics** [17]	England and Wales/2012	ICD-10 code: B02 and sub-codes for underlying cause of death	Age-specific incidence

### Characteristics of studies and databases

#### Published studies

All the studies used databases to identify HZ-related deaths (Table 3). Five studies used national statistics databases that collected data from death certificates for the general population [21,23,27,30,31]. Four of the remaining studies used national hospital statistics databases, two used both types of database and the last used data from a drug safety register, BIOBADASER, that monitors patients with rheumatic diseases treated with biological agents in 100 Spanish health centres [2,22,24-26,28,29]. Three studies reported mortality data specifically for patients with underlying conditions [26,27,29]. Four studies presented mortality data for deaths with HZ as the primary cause, one with HZ as the primary diagnosis at hospital admission and three with HZ as primary or associated cause; the remaining four studies did not specify if HZ was the primary or associated cause. Five studies used International classification of diseases-10 (ICD-10) codes and five used ICD-9 codes to identify HZ-associated deaths. The study in Spain using a drug safety database was the only one that reported the use of microbiological confirmation of infection to diagnoses HZ [29]. For the other studies, no information on the use of microbiological confirmation of the HZ diagnosis was provided. Some hospital-based studies reported underlying conditions: one in France reported that 22% of patients had ≥1 underlying conditions and another in Spain reported that 25% of the patients were immunocompromised but they did not provide mortality data for these patients separately [25,27]. Eight of the studies provided age-specific HZ-associated mortality data [2,22,24-28,30].

**Table 3:** **HZ mortality data from included studies**

**Study (ref)**	**Country**	**Period**	**Age (years)**	**Age at HZ-related death**	**Mortality incidence/100 000 (95% CI)**	**Case fatality rate (%)**	**Hospital fatality rate (%)**	**Number**
**Bilcke 2011** [21]	Belgium	1990-2007	60-70		0.06			
			70+		0.63			
**Gonzalez Chiappe 2010** [27]	France	2000-2007	Overall	96.8% (1360; 170/year) in those aged ≥65 years	0.286 (0.244-0.328)	0.075	2.012	1 405 (176 ± 13/year)
			≥65			4.36 ± 0.20		
			45-54		0.031 (0–0.069)	0.007		
			55-64		0.072 (0.009-0.135)	0.012		
			65-74		0.236 (0.102-0.370)	0.027		
			75-84		1.25 (0.90-1.60)	0.127		
			85-94		7.24 (5.66-8.82)	0.672		
			≥95		19.48 (11.50-27.47)	1.356	HFR: Malignancies: 4.236 (449/10 599) HIV/AIDS: 0.357 (26/7 277)	
							Diabetes: 1.654 (94/5 682)	
							Blood dyscrasias: 3.907 (82/2 099)	
							RA: 1.759 (38/2 160)	
							Malnutrition: 6.196 (64/1 033)	
							Organ transplant: 0.049 (1/2 023)	
**Ultsch 2011** [30]	Germany	2007-2008	50-54	73% (n = 48) deaths in ≥80 years	0.02 (0.00-0.10)	0.003		1
			55-59		0.00 NA	0		0
			60-64		0.02 (0.00-0.13)	0.003		1
			65-69		0.06 (0.01-0.17)	0.005		3
			70-74		0.11 (0.04-0.27)	0.010		5
			75-79		0.30 (0.14-0.57)	0.025		9
			80-84		0.54 (0.28-0.94)	0.043		12
			85-89		1.20 (0.67-1.98)	0.096		15
			≥90		3.86 (2.36-5.96)	0.293		20
			Overall		0.21 (0.16-0.26)	0.022		66
**van Lier 2010** [31]	Netherlands	2000-2007	≥60	92% deaths occurred in ≥ 75 years	0.59 (estimated with pop ≥60 from Eurostat)			Mean 18 deaths/year (range 13–26)
**de Melker 2006** [23]	Netherlands	1996-2002	≥75	97% occurred in ≥60 years	1.92 (estimated with pop ≥75 from Eurostat)			Mean 18 deaths/year (range 13 to 26)
**Mesquita 2013** [28]	Portugal	2000-2010	50-59	76.8 years (SD = 13.6)			1.0	17 (total)
			60-69				0.4	
			70-79				0.8	
			80-89				2.9	
			90-99				3.8	
**Perez-Sola 2011** [29]	Spain	2000-2006						0
**Gil 2009** [24]	Spain	1998-2004	50-59		0.3		3.8	156/year (mean)
			60-69		0.6		3.7	
			70-79		1.5		4.5	
			≥80		3.9		7.1	
**Gil-Prieto 2011** [26]	Spain	1998-2004	50-59				3.0*/1.1	
			60-69				2.7*/1.7	
			≥70				5.3*/2.7	
			50-59				No comorbidity: 1.1	
							Comorbidity: 3.0	
							Diabetes: 3.0	
							COPD: 4.2	
							CVD: 3.7	
			60-69				No comorbidity: 1.7	
							Comorbidity: 2.7	
							Diabetes: 1.9	
							COPD: 2.7	
							CVD: 3.4	
			≥70				No comorbidity: 2.7	
							Comorbidity: 5.3	
							Diabetes: 5.0	
							COPD: 5.0	
							CVD: 5.9	
**Gil-Prieto 2012** [25]	Spain	1997-2008	First diagnosis					
			≥50§				2.51§ (1.42-3.61)/	20
			≥50#				1.62# (0.67-2.5)	11
			50-59					
			60-69				0/0	0§/0#
			≥70				1.16§ (0–0.76)/0#	2§/0#
							3.61§ (1.97-6.25)/	18§/11#
							2.48# (1.03-3.93)	
			All ages					22§/11#
							1.89§ (1.1-1.27)/	
							1.22# (1.9-5.0)	
			Any diagnosis:					
			≥50					117§/70#
			50-59					4§/1#
			60-69					16§/3#
			≥70					97§/66#
**Brisson 2003** [22]	England & Wales	1991-2000	45-64$		0.014	0.002	0.612	2$
			≥65$		0.566	0.061	3.190	47
			all ages$$		0.094	0.031	2.722)	49
**Edmunds 2001** [2]	England & Wales	1995-1999	≥60		2.0 – 4.0			
			50-54			0.009		
			55-59			0.009		
			60-64			0.027		
			65-69			0.027		
			70-74			0.035		
			75-79			0.092		
			80-84			0.487		
			≥85			2.018		

*in those with diabetes, COPD and cardiovascular; § all; #immuno-competent; $data from national statistics; $$ HES data base.

#### Databases

We identified four databases that provided age-specific HZ mortality data (Table 2) [15-18]. Three of these databases were country-specific (France, Germany and United Kingdom (England and Wales)); the fourth database provided data for all European countries. As the national databases all contributed their data to the European database, we did not analyse their data, but extracted the data from the European database. This European database used International Statistical Classification of Diseases and Related Health Problems 10th Revision (ICD-10) code (B02) for all countries included in this review, except for Greece, which used ICD-9 codes, but there was no HZ mortality data available for Greece. We extracted HZ mortality data corresponding to people aged ≥50 years on 7 March 2014.

### HZ-associated mortality

#### Overall and age-specific incidence rates

**Figure 2:**
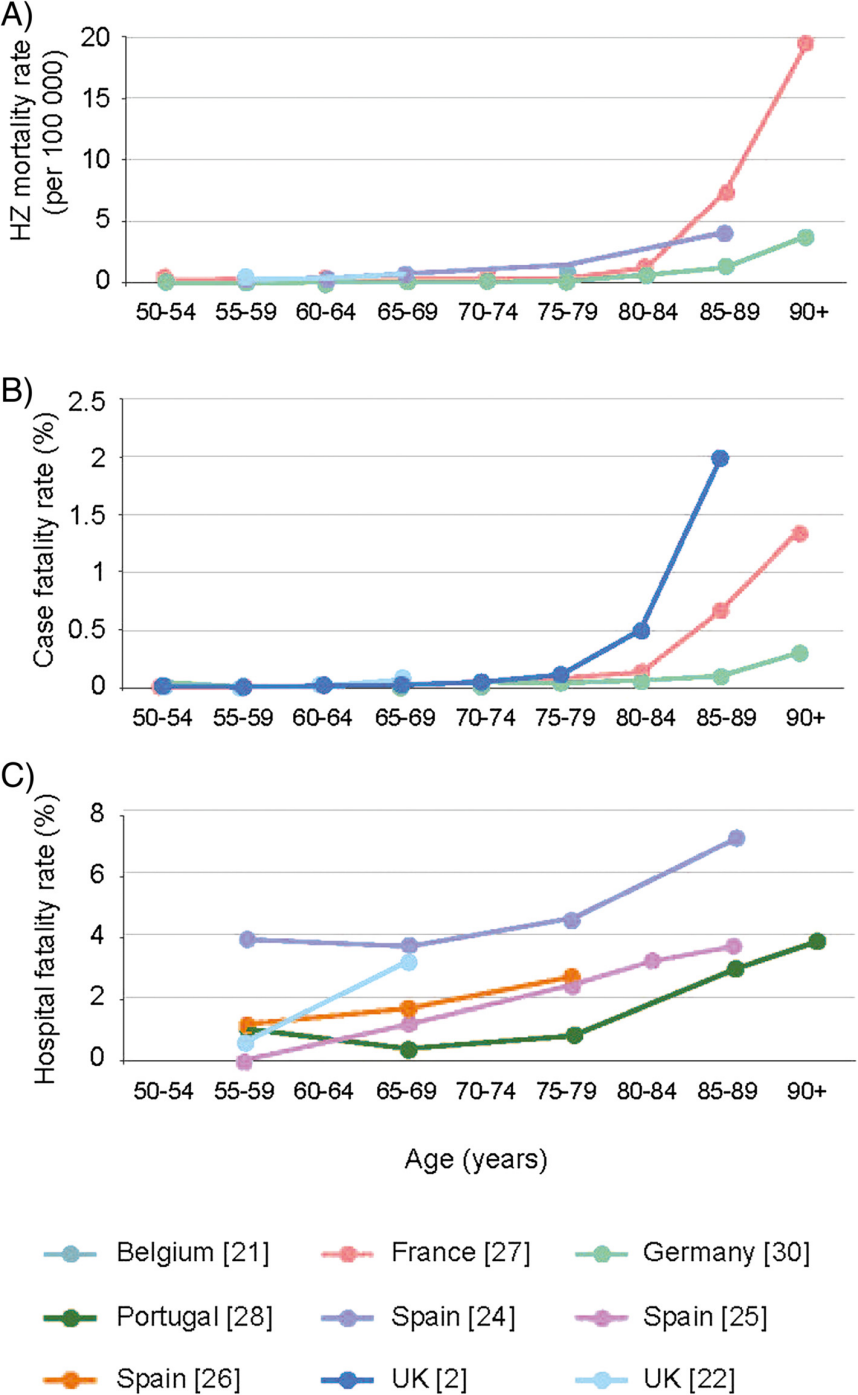
Mortality data from published studies: **A)** HZ mortality rate by age (years); **B)** HZ case fatality rate by age (years); **C)** HZ hospital fatality rate by age (years).

Eight of the studies provided HZ-mortality rates or provided data that could be used to estimate the mortality rates [2,21-23,27,28,30,31]. The age groups used were different between the studies but the overall trend was always towards a higher mortality incidence rate in the older age groups (Figure 2A). The mortality rates ranged from 0 in those aged 55–59 years in Germany to 19.5/100 000 for those aged ≥95 years in France [27,30].

**Figure 3:**
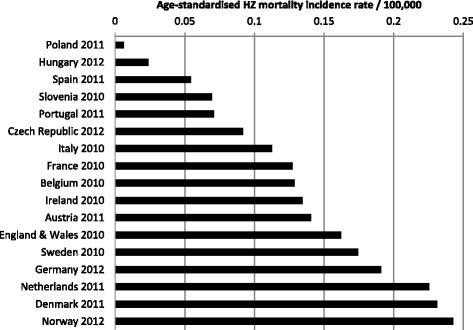
Age-standardized HZ mortality rate per 100 000 in all adults aged ≥50 years [15].

**Figure 4:**
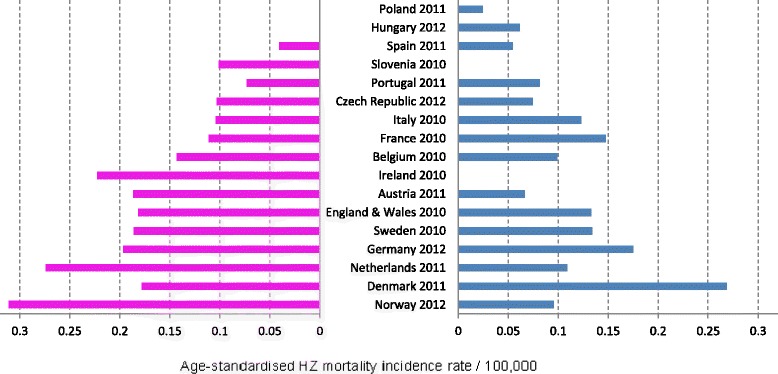
Age-standardized HZ mortality rate per 100 000 in females (left) and males (right) aged ≥50 years [15].

The data from the World Health Organisation (WHO) database showed that the estimates for the overall HZ mortality incidence in those aged ≥50 years varied widely between the countries for which estimates were available in 2011, ranging from 0.002 in Poland to 0.070 per 100 000 in Denmark, with a median of 0.039 per 100 000 (Figure 3) [15]. In countries where data are available for >1 year, although there were variations over time there did not seem to be any particular increasing or decreasing trends in the HZ mortality rate overall or for those aged 50–69 or ≥70 years (data not shown). The HZ mortality rate varied by gender and was generally higher in women, although in Spain, Italy, France and, particularly, Denmark, the mortality rate was higher in men (Figure 4).

**Figure 5:**
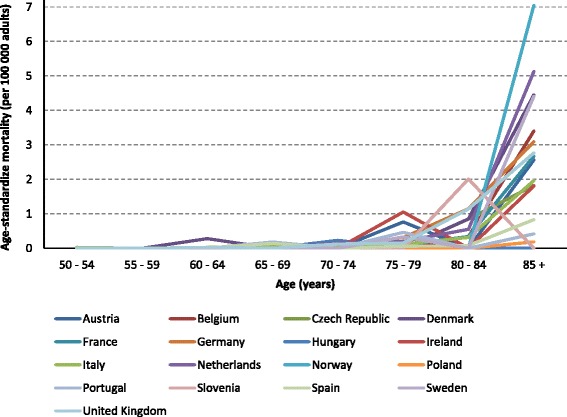
Age-standardized HZ mortality rate per 100 000 in adults by age group in the European countries [15].

Despite the between-countries differences in rates, there was a trend towards higher mortality rates in older age groups in all countries, with the increase occurring from the age of 70–74 years (Figure 5).

#### Age-specific case fatality and hospital fatality rates

Eight of the studies provided data to be used to estimate age-specific CFR (3), HFR (4) both (1) [2,22,24-28,30]. The age groups analysed varied between the studies but the overall trend was towards much higher rates in older age groups. The studies from UK provided estimation of CFRs that were around 2/100 000 in those aged 45 to 65 years old; and 61/100 000 in those aged ≥65. The CFR was available in four studies and varied from 0 in those aged 55–59 years in Germany to 2.02% in those aged ≥85 years in England and Wales (Figure 2B). The HFR was available in five studies and varied from 0.4% in those aged 60–69 years in Portugal to 7.1% in those aged >80 years in Spain (Figure 2C). The HFRs reported in Spain were higher than in other countries where the HFR was generally under 1% until the age of 75 to 85 years. Data from the two studies that provided fatality rates for sub-groups of patients with underlying conditions showed that there was a higher fatality rate in these populations [26,27]. In one of the studies, the fatality rate was also reported to increase with age in these populations [26]. One study in patients with ophthalmic HZ reported HFRs of 2.5% and 1.6% in those aged ≥50 in the overall population and in immunocompetent patients, respectively [25].

## Discussion

The results from this review show that HZ-associated mortality rate is generally low and, that despite the data being heterogeneous, in all studies mortality increased with age. Globally HZ-associated mortality is very low (<0.1/100 000) in those aged under 70 to 75 years and increases in older people, reaching between 1.2 to 7.2/100 000 in those aged ≥85 years. Not all studies reported trends in HZ mortality incidence over time, but one study in England and Wales reported a reduction in HZ-associated mortality over the 90's [22]. The overall EU data from the Detailed Mortality Database (DMDB) showed variations over time for those aged 50–69 years and those aged ≥70 years. However, the HZ mortality rate did not appear to increase or decrease from 1994 to 2012.

Similar to the EU data reported here, data from the US, for 1979 to 2007, showed that the HZ mortality rate was higher in older patients; an average of 134 deaths were recorded with HZ as the underlying cause, and 45% of these occurred in those aged ≥85 years [33]. The overall age-adjusted HZ-mortality was reported to decrease by 42% from 7.8 to 4.5 per 100 000. In another study in the US, from 1986 to 1995 the overall HZ-HFR was 5.3%; in those with underlying conditions the HFR was 8.7%, compared with 3.7% in those without [34]. In Australia, it was also reported to be higher in older people and women; 219 of the 238 (92%) deaths with HZ as the primary cause recorded from 1971 to 1993; (219) were in people aged >65 years and 65% were women [35]. Also the overall crude HZ mortality incidence was stable over this period (0.068 per 100 000); 0.043 and 0.092 per 100 000 in men and women, respectively. In another study in Australia, from July 1998 to June 1999 the overall HZ-HFR was 4% (186/4718) [36]. The HFR was higher in those with HZ as a secondary diagnosis compared with those with HZ as a primary diagnosis: 6% vs. 1%.

Mortality rates were higher when HZ was reported to be an associated or secondary cause of death suggesting that the HZ episode could have an indirect role in mortality [37]. Underlying comorbidities can increase the severity of the HZ episode and thus the risk of mortality [24]. It has been estimated that the risk of dying was five-times higher within three years of HZ reactivation than in age-matched controls that had not had HZ [27]. Diagnosis of HZ infection can increase the risk of subsequent diagnosis of diseases such as cancer, cerebrovascular disease or myocardial infarction and HIV [38-40]. In addition, hospitalisation of elderly people for acute diseases can result in functional decline and loss of autonomy [41-43].

It is important to be cautious when making comparisons between countries given the differences in study methodologies and coding practices in each country. Also, the data are presented for differing age groups, some of which are very large, making comparisons between studies difficult. In particular, some data are given for patients aged ≥65 years when we know that HZ-associated mortality rates are very different between those aged 65 to 75 years and those aged ≥85 years.

The differences in case definitions used could account for a substantial part of the heterogeneity. Most studies used clinical diagnosis for HZ, without requiring laboratory confirmation. In an elderly population, with many patients presenting underlying conditions or other comorbidities, it is difficult for a physician to be certain about the causal relationship between HZ and death, particularly if the certifying physician does not have access to all the patient’s medical records and autopsy results.

It is possible that the variations in the HZ-HFR are due to differences in the numerator since HZ hospitalisation rates may vary between countries due to different healthcare pathways, healthcare seeking behaviours, or perception of symptoms. In addition, differences in coding practices could contribute to this heterogeneity. Although only four studies in this review reported CFRs, there was less heterogeneity observed; it has been reported that HZ incidence rates do not vary between European countries [13]. Other factors such as differences in the prevalence of underlying risk factors for severe illness, differences in comorbidity rates or some other unidentified factors in different populations may also contribute to the observed heterogeneity.

Although most studies used HZ codes on death certificates for the primary cause of death, some used the codes for either primary or associated causes of death. Some of the limitations of using death certificates for estimating mortality incidence include incompleteness and errors. In the US, an assessment of the hospital records for decedents, whose death certificate recorded HZ as the underlying cause of death, showed that HZ was the underlying cause in only 52.5% (21/40) of cases and a contributing cause in another 12.5% (5/40) [33]. Other studies have reported that use of electronic or paper death certificates can lead to underestimations or overestimates of the true mortality rate due to other infectious and non-infectious diseases [44-47].

The strengths of our review include the fact that many studies and databases provide mortality data from national databases thus providing data for the whole population. However, there are some limitations. For example, the data are not from patient-level longitudinal prospective studies and, therefore, the retrospective design of the studies could reduce the accuracy of the data and provide an underestimation of the burden of HZ mortality. Some studies only reported mortality with HZ as the primary cause, whereas it has been suggested that the mortality is higher when HZ is an associated or contributory cause of mortality [36]. In addition, there were not much data available and there was a range of methods used for the estimations.

Six European Union countries have national surveillance systems and five have sentinel systems for HZ [32]. In the future, access to these surveillance databases should improve the quality of data concerning the incidence of HZ in Europe. In addition, the recent initiatives to harmonise coding for death certificates within Europe should play an important role in future HZ surveillance programmes, in particular by improving the comparability of data sources [48]. The initiatives are important for the evaluation of the impact of infant varicella vaccination and adult HZ vaccination on HZ incidence and mortality rates.

## Conclusions

In conclusion, although the available data on HZ-associated mortality are too heterogeneous to allow inter-country comparisons, they demonstrate that the mortality rate for HZ is not high, especially in younger people. However, mortality rates increase with age, particularly in those aged ≥75 years who often have underlying diseases and are at risk of functional decline and loss of independence if they are hospitalised.

## Abbreviations

CFR: Case fatality rate
DMDB: Detailed Mortality Database
EU: European Union
HFR: Hospital fatality rate
HZ: Herpes zoster
ICD: International classification of diseases
MeSH: Medical Subheading
PHN: Post-herpetic neuralgia
VZV: Varicella-zoster virus
WHO: World Health Organisation
